# Perceptions of patient aggression in psychiatric hospitals: a qualitative study using focus groups with nurses, patients, and informal caregivers

**DOI:** 10.1186/s12888-022-03974-4

**Published:** 2022-05-18

**Authors:** Maritta Välimäki, Tella Lantta, Yuen Ting Joyce Lam, Teris Cheung, Po Yee Ivy Cheng, Tony Ng, Glendy Ip, Daniel Bressington

**Affiliations:** 1 Xiangya School of Nursing, Xiangya Center for Evidence-Based Practice & Healthcare Innovation, Central South University, 172 Tongzipo Road, Changsha, 410013 Hunan China; 2 Department of Nursing Science, ICT-city, University of Turku, 20014 Turku, Finland; 3 School of Nursing, The Hong Kong Polytechnic University, Hung Hom, Kowloon, Hong Kong (SAR), China; 4 Community Psychiatric Services, 12/F, Block A, Pamela Youde Nethersole Eastern Hospital, Hospital Authority, Hong Kong (SAR), China; 5 Department of Psychiatry, Pamela Youde Nethersole Eastern Hospital, Hospital Authority, Hong Kong (SAR), China; 6 Central Nursing Division, Kwai Chung Hospital, Hospital Authority, Hong Kong (SAR), China; 7 College of Nursing and Midwifery, Charles Darwin University, Ellengowan Drive, Casuarine, Darwin, NT 0909 Australia

**Keywords:** Focus group, Hospital, Informal caregiver, Nurse, Patient, Patient aggression, Psychiatric, Qualitative, Violence

## Abstract

**Background:**

Aggression in psychiatric hospitals has been of interest to researchers. Information on how different stakeholders perceive patient aggression remains equivocal. Even less is known about possible similarities or differences in stakeholders’ perceptions of how aggressive behaviour is understood, managed and prevented in psychiatric hospitals. We aimed to explore multiple viewpoints on patient aggression, its possible causes and outcomes, and development ideas for prevention and management.

**Methods:**

A qualitative design was adopted. The data were collected using focus group interviews. A thematic approach was used for interpretation. The data were collected on 15 adult wards in two inpatient psychiatric settings in Hong Kong. Participants were nurses working on the psychiatric inpatient wards, patients admitted to the wards, and informal caregivers visiting inpatient wards (*N* = 94).

**Results:**

Commonalities between all groups were found on how patient aggression is perceived, and why it occurs. Patients and especially nurses described how patient aggression occurred with no clear reason or forewarning and how patients were physically controlled or restricted after aggressive events. Only nurses and patients expressed experiencing physical burden, while all groups considered psychological burden to be a consequence of aggression. All groups proposed that helpful attitudes among nurses, better communication, structural changes, and better self-management skills would prevent patient aggression. Risk assessment was proposed only by nurses and patients, while safety measures were proposed by nurses and informal caregivers only. The use of restrictive interventions to manage aggressive events was proposed by all groups.

**Conclusions:**

Despite the complex diversity of perspectives in different stakeholder groups regarding patient aggression, the findings highlighted that it is possible to achieve some mutual understanding of aggression in psychiatric hospitals and identify areas to be developed. Staffs’ attitudes and skills for engagement and communication with patients and informal caregivers should be improved. There is also still room to develop the therapeutic environment and culture toward meaningful activities during the treatment period.

**Supplementary Information:**

The online version contains supplementary material available at 10.1186/s12888-022-03974-4.

## Background

Aggressive behaviour in closed institutions is a global concern [1, 2]. It causes several negative effects on staff members involved, such as physical injuries [3], decreased job satisfaction [4] and even death [3]. There is a consensus that the aggression phenomenon is multidimensional, and the picture concerning the complex interplay of causal variables and their respective impact is still unclear [5]. Aggression can be defined broadly as any behavior intended to harm another person. It can be conceptualized as reactive (a response to perceived threat that is impulsive and emotionally charged) or proactive (premeditated and controlled) [6]. It can also be understood as a continuum of severity, where agitation evolves into aggression and ultimately into violence [7].

The factors associated with patient aggressive behaviour are heterogenous [6]. The phenomena of aggression is contextual and relational, and therefore relationship between risk factors and aggression is not straightforward [8]. Typically, patient aggression in psychiatric hospitals is described using quantitative approaches regarding prevalence or associated risk factors [6] including sex, age, comorbid psychiatric disorders, socioeconomic status, and history of aggressive behavior [9]. Staff related and environmental risks factors for aggression have also been identified using quantitative methods [7]. Current explanation of reasons for aggression is a combination of factors embodied in patients, staff and the ward environment [7]. On the other hand, aggressive inpatient incidents have not been adequately explained in research [10]. When management of aggression is a focus of treatment, opinions for aggression should be shared from different points of view, including those of clinicians, patients, and families [8].

A growing body of evidence has used qualitative approaches to understand experiences related to aggression [5]. Studies have shown that nurses see patient violence as an outcome of their mental status and that violence therefore cannot be avoided; it is part of nurses’ job [11, 12]. Patients themselves think that environmental triggers and interpersonal relationships can lead to aggression [11]. Further, family members experience patient aggression as burdening and traumatizing [13]. Still, little interest has been shown in understanding aggressive behaviour by combining multiple perspectives using qualitative methods [14, 15]. Cornaggia et al. [16] have identified a disagreement between patients and staff concerning the predictors of aggressive episodes, patients tend to emphasize to a greater extent the significance of environmental conditions and poor communication, while staff tend to rely on internal variables like the patient’s illness as the main reason. Gudde et al. [5] conducted a systematic review and thematic synthesis of qualitative studies based on users’ experiences. They reviewed 13 studies (ten qualitative and three mixed methods) and showed that aggressive incidents are triggered when users experience staff behavior as custodial rather than caring and when they feel ignored. Patients clearly articulated important insights gained from their experience and expressed a strong desire to be more involved in questions regarding how to define, understand, prevent, and manage aggressive situations effectively.

We also systematically searched and identified nine qualitative studies that described both patient, staff and/or caregiver perspectives in a same study on aggression in psychiatric hospitals. Only one of them included family members (*n* = 4 persons in individual interviews) and this study used group format interviews (only for staff) [17]. In a study by Wright et al. [18], patients in security units felt that staff had negative attitudes towards them, which potentially caused aggressive situations. On the contrary, staff thought that they were doing their best to bring patients and nurses closer together. In a study by Goodman et al. [17], staff expressed that patients can be labelled as ‘mad, bad or sad’. Especially aggression from the perspectives of patients diagnosed with personally disorder could be responded to in negative, unempathic ways. On the contrary, patients and family members criticised power and control over patients, which was seen by setting unnecessary limits on patient behaviour. Aggression management practices were not well-accepted by patients, as staff may have used heavy-handed approaches to escalating aggression, causing fear and traumatisation [17]. On the other hand, harsh handling of aggressive patients was reported by both staff and patients in a study by Pelto-Piri & Kjellin [ 14]. Vermeulen et al. [19] interviewed patients and nurses after aggressive incidents and found that the views of patients and nurses were similar regarding the facts of the incidents that had occurred. On the other hand, patients considered the incidents to be less severe than how nurses viewed them. Another discrepancy in the data was that patients proposed more practical and treatment-oriented solutions than nurses did.

To better understand inpatient aggression, a combination of different stakeholders’ perceptions is important as aggressive behaviour can be seen differently from various viewpoints [11]. Engaging different stakeholders, including patients, can help in the establishment of a culture of safety to improve the quality of inpatient psychiatric care [20]. The risks for aggression, for example, should be interpreted and discussed with the patient because patients themselves can provide their views on etiology, triggers, and contributors to aggression. Patients’ families should not be ignored either because of their knowledge and experience of being with the patient [8]. To our knowledge, however, previous research has shed only limited light on aggression in psychiatric hospitals by combining the perceptions of various stakeholders using qualitative methods. Studies comparing perceptions of patient aggression in psychiatric hospitals using focus group interviews and a qualitative approach to combine the viewpoints of patients, nurses, and informal caregivers are also lacking. If a shared understanding of aggression is missing, prevention and management efforts are not viewed in the same way [14, 21].

This is the first study ever where qualitative methods are used to describe multiple viewpoints of nurses, patients and informal caregivers, on patient aggression. We will describe from the points of nurses, patients and informal careers, what happened in aggressive events in inpatient psychiatric hospitals, what are possible causes of the aggressive events, outcomes, and development ideas for prevention and management of these events. The knowledge gained in this study could help share the commonalities and differences regarding how patient aggressive behaviour is perceived, a perception that still lacks consistency, to find areas to be developed in the future. The topic is important as a comprehensive conceptualization of aggression in inpatient mental health settings is still needed [22]. This focus group study is a part of the series of sub-studies to understand physical restriction practices with psychiatric patients in Hong Kong (1-ZE84).

## Methods

### Aim

To explore multiple viewpoints on patient aggression, its possible causes and outcomes, and development ideas for prevention and management from the point of view of nurses, patients, and informal caregivers based on focus group interviews.

### Design

This study uses a descriptive qualitative study design. The qualitative design is appropriate to our study because we aimed to explore participants’ beliefs, experiences and motives that explain why the specific behavior, aggression, occurs [23]. We used a naturalistic perspective to examine a phenomenon of aggression in its natural state [24]. The philosophical assumption of the study relied on the interpretivism paradigm, which propose that participants have their own experience of reality to gain a better understanding of phenomenon through the experiences of those who have directly experienced the phenomenon [25]. This assumption guided our interview questions and the data categorisation in two ways. First, the interviews included a limited number of open-ended questions allowing participants to express their thoughts freely. Second, the data were analysed inductively without a theoretical framework used as pre-determined codes or categories [26].

The reporting of this study complies with the consolidated criteria for reporting qualitative research (COREQ) recommendations [27].

### Setting

The data were collected in inpatient hospitals in Hong Kong, China (SAR) that represent typical hospital clusters in Hong Kong. All seven hospital clusters were invited to join the study, and two clusters joined after the information session was organized. Adult wards in the hospitals were selected as study sites as physical restrictions are regularly used there to manage aggressive events. In total, 15 wards in two hospitals participated in the study, including both acute admission and rehabilitation wards for adult patients. The size of the wards ranged from 18 to 90 beds.

### Participants and recruitment

We recruited participants to join the study on the adult wards where patient aggression typically occurred and physical restrictions were used to manage aggressive events. We recruited nurses working on these study wards, patients who were admitted to the wards, and informal caregivers of the patients (e.g., family members, relatives, friends) who visited on the study wards, to join the study. We assumed that these groups have some experiences of aggressive events because of the type of their hospital setting. Therefore, they could offer comprehensive information on this controversial topic based on their own experiences and offer a rich variety of perceptions [28].

Purposive sampling was used to recruit participants to capture the diversity of perceptions and ensure a broad representation of individuals in each group [29]. The ongoing study was advertised on wards using study posters and leaflets. Individuals were eligible to join the study if they felt they were able to provide first-hand experiences regarding the nature, causes, outcomes or possible preventability of aggressive events in the settings. More detailed eligibility criteria for specific groups were as follows. First, nurses with different education levels were invited (registered nurses, enrolled nurses, or assistant nurse). A mixture of professions and work experiences ensures a variety of perceptions on the interview topic [30]. Nurses were recruited by the Head Nurse who acted as a contact person for the study. We excluded nurses who did not work in direct patient care or those with a part-time contract or did not accept audio recording. The Head Nurses screened nurses on the wards based on the study criteria and invited those who fulfilled the criteria. Second, patients had to be at least 18 years old, could be either sex, able to speak and/or read Chinese (Cantonese), be willing to participate and share their views in the group, and accept that there would be audio recording. Patients were invited to participate in the interviews only if they were assessed as mentally capable for interviews (assessed by nurses and confirmed by the chief psychiatrists). And third, all informal caregivers visiting the study wards were considered suitable for the focus group interviews and were invited to participate in the study. At both hospitals, informal caregivers were allowed to meet patients under the supervision of staff members, during a specific time period (2 hours per day) in room dedicated for outside visitors only. The informal caregivers had to be at least 18 years old, could be either sex, and had to be willing to participate and share their views in the group, and had to accept that there would be audio recording. Patients and informal caregivers were recruited by the Research Assistant (RA) who spent time on the study wards at the time of the recruitment and the data collection periods.

The sample size estimations were made based on assumptions in the literature: about two focus groups in each target group (with 2 to 10 participants in each group) are sufficient for reaching data saturation, i.e. no new information or themes emerging in the data [31], and about 48–60 participants ensured meeting the research aims [32].

### Data collection and interview template

Data were collected in focus groups, a valuable method for obtaining a range of perceptions and experiences of a studied phenomenon [30]. Focus group data collection was selected over individual interviews because focus group interviews have been considered to be one of the most appropriate methods for capturing opinions using a reflective process facilitated in social interaction. In this sense, the key feature in focus group is the active interaction among participants to explore their views and opinions. Compared with other data collection methods, it provides insights into the sources of complex behaviors and motivations [33], which is in line with a phenomena of aggression [5]. The interviews were conducted in the Cantonese language. The interview topic, patient aggression, was first discussed in larger groups at the study hospitals. Aware of the sensitive nature of the topic, we organized separate groups for nurses, patients and informal caregivers where each group member was encouraged to discuss specific interview questions regarding aggression, which is part of daily life in many hospital wards. Participants’ experiences were not matched with specific events.

All main facilitators (females/males; LFW, MHC, ZC and YTJL) were members of the research team (associate or assistant professors, or researchers) specialized in psychiatric care (registered nurses with PhD, for example). The most experienced facilitator led the focus group discussions as the main facilitator, and trained co-facilitators were responsible for recording and writing notes (two facilitators in each group). For practical and security reasons, availability of staff members was ensured for each interview. Focus groups were audio-recorded with the permission of the participants.

The data were collected using focus group interviews on the hospital premises. At the beginning of the interview, facilitators introduced themselves, participants received more detailed information about the study (purpose, aims, goal of the interview) and information about practical arrangements and ethical issues (e.g., voluntary participation, confidentiality). Participants were assured that they could withdraw from the study at any time without consequences. To decrease any pressure and encourage information sharing freely during interviews, participants were ensured that they would not be judged for their opinions and that their contributions were vital to the study. The participants were informed that if they had any further questions or worries after the interviews took place, they could contact a designated person on their ward with whom they could discuss their concerns. Non-participation were not recorded for the confidentiality reasons. Written informed consent was obtained before enrolment and background characteristics were collected. For nurses, patients and informal caregivers, sociodemographic characteristics were collected (age, sex, education level, marital status).

An interview template with open-ended questions tested in other studies was used [34]. Open-ended questions were used to encourage each participant to describe their experiences or perceptions using their own words [27]. The content of the questions were the same in each group: 1) Describe patient aggressive behaviour on the ward—what has happened? 2) What might be reason for an event? 3) What are the consequences for such an event? and 4) How could aggression management be developed?

The original template for the focus-group interviews has been added as supplementary material (Supplement 1). By following the focus-group template, the duration of the interviews was 40–84 minutes (average 61.25 minutes). No repeated interviews were carried out. The interviews were transcribed into 458 pages (Word program, line space 1.5).

### Data analysis

The thematic analysis process by Braun and Clarke [35] was followed with an inductive approach. This allowed us to capture the ‘voices’ of the participants in the data set instead of identifying specific codes based on theories or models. Themes in the analysis process were formed in text data in the Cantonese language.

**Fig. 1:**
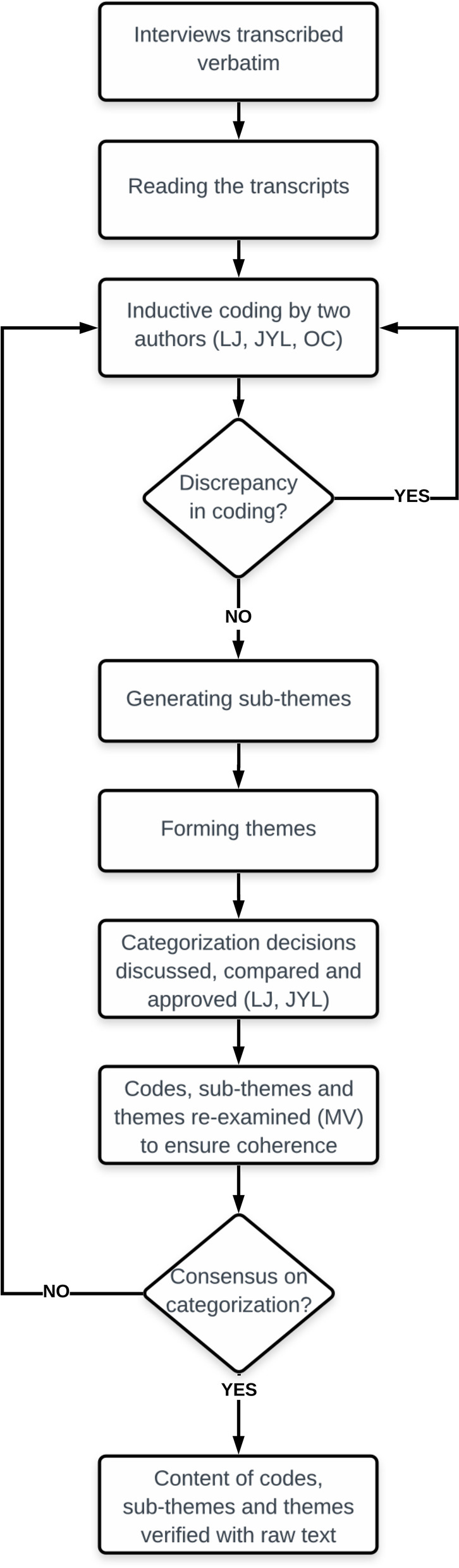
Coding tree of the qualitative data

First, the entire dataset was read carefully. Second, specific codes were formed using words, phrases or sentences that would help meet the research goal. To ensure the validity of the coding process, the data were coded separately by two persons (LJ/YTJL/OC). If any discrepancies were identified in the coding results, the whole data set was re-coded. Third, the preliminary codes were collated into sub-themes according to inductive patterns in the data set: similar content was combined to form sub-themes. Fourth, the content of the sub-themes was further compared and combined into specific themes. After each categorization phase, the data categorization was discussed, compared and approved by two persons (LJ, YTJL). To enhance the trustworthiness of findings, the analysis process was re-examined by one author (MV) to ensure coherence of the codes within each theme (Fig. 1).

Robust study methodology was ensured in the data collection, analysis, and interpretation of the results using approaches recommended by Kidd & Parshal [36]. For the data collection, all interviews were recorded to ensure verification of the discussion. Three to four researchers and/or staff members joined in each group to make notes in case of lively discussions and note non-verbal communication. Equivalence between groups was ensured by training the group facilitators and maintaining interview procedures same across groups. Most of the focus group facilitators also transcribed the audio recordings, which facilitated the checking of the transcripts against the tapes. Regarding the data analysis, analytical approaches were kept flexible to identify any important influence of the group or individual participant. Therefore, opinions of individual participants were seen as important as a consensus opinion made by the group. Conflicting viewpoints were included in the analysis and no ‘best’ responses were selected. If discussions included topics containing large, discourse chunks, such material was subjected to a more detailed analysis, and different level of coding was used in one theme. In addition, all data were coded by two persons and the equivalence of coding was ensured by randomly selecting test coding pages. The internal consistency of coding was enhanced as 1–2 team members did the primary coding, and the results were checked by the third person. Further, validity of the coding was ensured by selecting those researchers with professional training related to mental health. In addition, two focus group participants validated the transcripts and native Cantonese-speaking persons categorized the qualitative data. The validity of the results was also ensured by two focus group participants who verified the results of the data categorization in the manuscript, and the preliminary results were shared between the team members who were working in the study settings. The research team were mindful of reflexivity issues throughout the analysis process, particularly in relation to how individual perceptions, experiences or world views may have influenced the analysis or interpretation of the results.

To ensure credibility of the study results in local context, we conducted member-checking to ensure that the research findings of the focus groups accurately reflected their lived experiences. This was done by organising a meeting to discuss the results with a group of staff members (patients and family members were not invited due to privacy reasons). In addition, a triangulation method was used by comparing and combining the results from the current study with the findings obtained from our larger study that used other data collection methods. The methods used were the analysis of local hospital documents guiding patient physical restriction practices in study wards [37]; a quantitative survey of nurses’, patients’ and informal caregivers’ attitudes toward aggression (submitted); and an analysis of the hospital register data regarding patient restrictions [38]. All these separate data sets confirmed the results of this focus group study and increased its credibility.

## Results

### Description of the data characteristics of the participants

Between 27 April and 11 July 2018, 12 focus groups (4–13 participants) were organized, six at each of the two hospitals, for a total of 94 participants (Table 1).

**Table 1:** Characteristics of the focus group participants (*N* = 94)

	Nurses *N* = 36	Patients *N* = 28	Informal caregivers *N* = 30
Age			
18–35	19 (53%)	13 (47%)	2 (7%)
36–50	9 (25%)	9 (32%)	3 (10%)
51–60+	8 (22%)	6 (21%)	25 (83%)
Gender			
Male	16 (44%)	12 (43%)	12 (40%)
Female	20 (56%)	16 (57%)	18 (60%)
Education			
No education	–	–	2 (7%)
Primary school	–	2 (7%)	6 (20%)
Secondary school	2 (6%)	18 (64%)	10 (33%)
Tertiary school or above	34 (94%)	8 (29%)	9 (30%)
Missing	–	–	3 (10%)
Marital Status			
Single	14 (39%)	20 (71%)	4 (13%)
Married	21 (58%)	6 (21%)	18 (61%)
Divorced	–	2 (8%)	4 (13%)
Widowed	1 (3%)	–	4 (13%)

### Description of aggressive events—what has happened?

The term *physical aggression* was described as action aiming to harm something or someone. *Verbal aggression* was described as the use of language or voice, for example, to provoke other people. *Threat* as an action was identified as using body posture, an object or behaviour with the intention of making people feel afraid. The term *target of aggression* was divided into three categories: other people (e.g., nurses, patients), objects (e.g., chairs, a wall), and the patient themselves. Themes and sub-themes, along with quotes, to describe the meaning of aggression are presented in Table 2.

**Table 2:** Description of the meaning of and reasons for aggression among nurses, patients and informal caregivers

**Themes**	**THE MEANING OF AGGRESSION** **Quotes from nurses, patients and informal caregivers**
**Type of aggression**	**Physical aggression** “A patient slapped the nurse and hit the staff member on the head.” (Nurse, Group 1) “I used to fight with other patients.” (Patient, Group 2) “A patient hits anybody, she would even hit the doctor, she hit the nurse before many times.” (Informal caregiver, Group 2)
	**Verbal aggression** “80–90% incidents are verbal aggression, like arguing, scolding…which means scolding somebody, provocation to fight.” (Nurse, Group 4) “A patient intended to say something to provoke others.” (Patient, Group 4) “A patient argued with the patient who spoke foul language.” (Informal caregiver, Group 3)
	**Threat** “A patient grabbed a chair, held it dangerously and walked around the visiting room.” (Nurse, Group 1) “[another patient] had aggressive posture.” (Patient, Group 4) “A patient provoked another patient to fight with some aggressive motions.” (Informal caregiver, Group 3)
**Target of aggression**	**Other people** “A patient punched a staff member’s chin.” (Nurse, Group 3) “A patient took a chair and threw…threw it in my face.” (Patient, Group 3) “A patient kicked a nurse.” (Informal caregiver, Group 2)
	**Objects** “A patient grabbed the chair and wanted to damage the lighting on the ceiling.” (Nurse, Group 3) “A patient kicked towards the table” (Patient, Group 1) “A patient expressed aggressive behaviours and threw things towards a nurse.” (Informal caregiver, Group 4)
	**The patient themselves** “A patient took out a cutler knife and intended to harm himself.” (Nurse, Group 1) “I wanted to commit suicide.” (Patient, Group 4) “A patient had suicidal thoughts.” (Informal caregiver, Group 3)
**Themes**	**THE REASONS FOR AGGRESSION** **Quotes text of nurses, patients and informal caregivers**
**Unstable mental status**	“The patient said he doesn’t know why…he saw…something made him afraid, then he attacked.” (Nurse, Group 4) “When the patient hit my head, I felt she was mentally unstable.” (Patient, Group 3) “I do not know, maybe the co-patient lost her emotional control.” (Informal caregiver, Group 1)
**Unmet needs**	“...the patient wanted a new pillowcase…a shortage of manpower at that time, but the patient felt annoyed and held up a chair in nurse station.” (Nurse, Group 1) “Because I felt that they (nurses) did not allow me to leave, I dashed to the main door.” (Patient, Group 4) “I want to leave”, the patient said. Then she dashed to the door and scolded staff with foul language.” (Informal caregiver, Group 3)
**Social conflicts**	“A patient banged his head on the wall…the patient felt unhappy because his mother said something to him.” (Nurse, Group 2) “Actually, I am not sure of the reason, but I guess, a patient showed his middle fingers to other patients” (Patient, Group 3) “The patient had some verbal conflict with a visitor, then they started to fight.” (Informal caregiver, Group 1)
**No clear reason**	“I was attacked by a patient from behind without any reason…” (Nurse, Group 1) “I do not know, it was all of a sudden, I was so scared. The patient slapped my face when I was applying lotion.” (Patient, Group 3)
**Themes**	**CONSEQUENCES OF PATIENT AGGRESSION** **Quotes text of nurses, patients and informal caregivers**
**Action**	**Seeking help** “…another nurse asked other patients to seek other nurses’ help...the nurse yelled for help…” (Nurse, Group 1) “Police were called.” (Patient, Group 2) “After seeing the doctor and adjusting the medication, the patient could become calm.” (Informal caregiver, Group 2)
	**Controlling** “…the patient was under restraint finally” (Nurse, Group 2) “Finally, she was under restraint on the bed and likely to be given sedation.” (Patient, Group 1) “My son was restrained on the bed. And I was told that I could not visit him because he was under restraint and his emotions were unstable. But finally I could visit him, a few nurses monitored when visiting him.” (Informal caregiver, Group 2)
	**Calming down** “After …after comfort and negotiation, the patient calmed down, no need to be under restraint.” (Nurse, Group 1) “The nurse rushed to help the victim patient and asked if he was OK, and then explained to him that he should not attack a patient on purpose.” (Patient, Group 1) “A patient kicked the bed badly. A few nurses rushed to handle it. The patient’s father also went in just about visiting time to hug his daughter and the patient could calm down. Finally, the patient was not restrained.” (Informal caregiver, Group 2)
**Burden**	**Physical burden** “I witnessed the waist of my colleague was hurt and his fingernail was hurt.” (Nurse, Group 2) “One of the patients was bleeding from the nose.” (Patient, Group 3)
	**Psychological burden** “The nurse was not severely hurt but very scared and cried…” (Nurse, Group 3) “…the patient was restrained for 2–3 hours on the bed due to fighting, felt very bad during restraint, did not feel calmed down.” (Patient, Group 4) “The nurse just held the patient down a bit but did not pull the patient back. This incident had made a psychological impact on a relative or another patient as a witness. He was worried about his wife who stayed in the same ward, he could not sleep that night…very worried.” (Informal caregiver, Group 3)

The participants expressed different feelings related to aggressive events. They were captured in the original data as follows:

“*The situation was a bit chaotic…but luckily no one injured…I felt worried, but luckily no one hurts. The seriousness depends on if there’s any negative consequences. Patients were also scared. The situation was chaotic…I was in panic”* (Nurse, Group 4)

“*I felt angry, so I fight.”* (Patient, Group 2)

“*I was scared. The situation was so dramatic…”* (Informal caregiver, Group 3)

### Reasons for patient aggressive behaviour

First, all three groups stated that the mental status of a patient can be a cause of patient aggressive behaviour. Participants identified features in patient illness, which reflect their inner world. Patients can become anxious or paranoid, hallucinate, or hear voices, which can make them aggressive. Second, all groups described patients’ unmet needs. Nurses described a variety of situations that spark anger in patients, such as a patient not being allowed to leave the ward. Patients and informal caregivers described situations in which patients were disappointed due to a lack of communication from staff or a sudden change in treatment without a clear explanation. Third, problems with communication and relationships can cause problems on the ward as all groups recognized social conflicts as a source of aggression. Patients eagerly identified conflicts with their peers.

Fourth, only nurses and patients described how patient aggressive events sometimes occur without any clear reason, for example, a patient suddenly pulling a doctor’s hair, becoming uncooperative, or behaving aggressively without any visible or rational reason. A commonality among the descriptions was that nurses were unaware of patient burden or were not prepared for the patient behaviour they encountered (Table 2).

Regarding feelings related to the reasons for aggressive events, the participants expressed a sense of helplessness: both nurses in Group 1 and relatives in Group 3 described aggression as ‘*unpredictable*’ and ‘*unforeseeable’*. Perhaps therefore nurses seemed to feel guilty, which can be identified in the following statement:

“*I will be more alert and careful in the future*.” (Nurse, Group 2). On the contrary, patients felt that they did what they had to do:

### Consequences of patient aggression

All groups described seeking help to manage aggressive situations (Table 2). Nurses frequently recounted seeking help from the police, security personnel or other staff. Patients and informal caregivers sought help more often through treatment, for example, a ‘doctor was consulted to adjust medication’.

Groups described a variety of actions used to physically control patient aggression. Beyond physical restrictions or sedation (‘going to the sleeping room’), patients shared their experiences of nurses offering a private room in which they could calm down, or nurses asking patients what had happened or what the reason had been for their behaviour, while informal caregivers described how family members had comforted patients after an event. Nurses described physical restriction methods used on patients after aggressive events. These included, for example, holding, separating a patient and a nurse, putting a patient in restraints or sedating a patient.

Regarding physical burdening, nurses described a wide variety of injuries based on patient aggressive behaviour, for example, a scar on the head from being bitten. Patients described pain or bleeding from the nose. Informal caregivers did not describe any physical injuries. As for psychological burdening, nurses reported that they had cried and been emotionally hurt, scared, or worried. Patients also expressed feeling bad after hours of being in restraint. Informal caregivers felt scared and worried after incidents of patient aggression.

### Development areas for the future

All three groups thought that healthcare personnel should have more of a helping attitude. Nurses emphasized the importance of guiding patients and perceiving their views to understand and satisfy their needs. Patients proposed that nurses should be more welcoming, show more understanding, be active in approaching patients, and be more flexible. Patients thought they should be allowed to show their emotions. Informal caregivers proposed that nurses should better understand patients’ problems, and provide more support and comfort; instead of depending on medication to solve problems, nurses should determine the reasons for aggressive events occurring (Table 3).

**Table 3:** Development ideas from nurses, patients and informal caregivers

Themes	DEVELOPMENT IDEAS Quotes text of nurses, patients and informal caregivers
**Helping attitude**	“...understand what the patient needs” (Nurse, Group 1) “I think ward staff should be more considerate towards patients.” (Patient, Group 1) “...nurses should...understand their problems, why they would do so” (Informal caregiver, Group 1)
**Communication**	“For prevention, firstly, to the patients, some education, like public education, or let them have more understanding about the ward, the routine, environment, facilities, let them know their rights and obligations, regulations.” (Nurse, Group 1) “When the nurse found that that patient started to behave differently, they would approach her, talk with her, find out the current condition…” (Patient, Group 1) “I support the idea that talking with patients is very important, more time to talk with the patients is highly recommended.” (Informal caregiver, Group 1)
**Structural changes**	“To improve the environment, a better environment for the patient. Now it is so congested having 60 patients on the ward and 5 nurses, a 1 to 12 ratio, what kind of care can be expected? Besides staying in such a full ward…how can one be happy?” (Nurse, Group 2) “Sleeping time could be longer. The sleeping bed is locked after 6.00…sometimes I feel sleepy after medication…actually taking a nap, would not disturb nurses…do not limit every patient’s sleeping time.” (Patient, Group 2) “There are not enough healthcare professionals to talk with my daughter, like not enough social workers, clinical psychologists to talk with, let her vent out the emotion…just apply restraint when unhappy…need to have more nurses or volunteers, etc. to talk with them.” (Informal caregiver, Group 2)
**Restrictive interventions**	“Any stimulant or anything leading to less aggressive behaviour should be reduced…” (Nurse, Group 2) “I think restraining the attacking patient on a bed would calm them down.” (Patient, Group 2) “Try not to provoke the patient, try your best to accommodate her, not to say something negative.” (Informal caregiver, Group 1)
**Self-management**	“Need to calm yourself down, emotion management… keep calm” (Nurse, Group 1) “I have to behave myself if I want to be discharged…if I have aggressive behavior, patients would be under restraint, more medication, delay the time of discharge.” (Patient, Group 1) “Try not to provoke, try your best to accommodate…” (Informal caregiver, Group 2)
**Assessment**	“Should regard that patient as an unexpected …patient, so when taking blood pressure with the patient, should be more alert that he would attack others at anytime.” (Nurse, Group 2) “I will be more cautious of patients’ facial expressions to enhance the alertness in the future.” (Patient, Group 1)
**Creative activities**	“…newspaper reading, art craft, exercise, iPad to spend time on, make life here less dull. Patients sitting here having different mental illnesses. If there are no activities for them to distract their thinking…would be easier to get more disorder of mind.” (Patient, Group 2) “I suggest adding like an exercise bike, a treadmill for some patients to use their energy, not to attack others.” (Informal caregiver, Group 2)
**Safety measures**	“For the patient with violent propensity, a security guard would follow the patient within a 3-m distance.” (Nurse, Group 1) “Security checks are needed to prevent inappropriate objects in the ward, this is important.” (Informal caregiver, Group 1)

In generally, all three groups shared the same feeling that a lot of things could be done to develop treatment environment for patients. 

### Comparison of perceptions between stakeholder groups

A summary of the themes from the different stakeholder groups is presented in Table 4.

**Table 4:** Comparison of themes among nurses, patients, and informal caregivers

	Nurses	Patients	Informal caregivers
**Aggressive events**			
***Type of aggression***			
Physical aggression	**X**	**X**	**X**
Verbal aggression	**X**	**X**	**X**
Threat	**X**	**X**	**X**
***Target of aggression***			
People	**X**	**X**	**X**
Objects	**X**	**X**	**X**
The patient themselves	**X**	**X**	**X**
**Reasons for patient aggression**			
Unstable mental status	**X**	**X**	**X**
Unmet needs	**X**	**X**	**X**
Social conflicts	**X**	**X**	**X**
No clear reason	**X**	**X**	
**Consequences of patient aggression**			
***Action***			
Seeking help	**X**	**X**	**X**
Controlling	**X**	**X**	**X**
Calming down	**X**	**X**	**X**
***Burdening***			
Physical burden	**X**	**X**	
Psychological burden	**X**	**X**	**X**
**Development areas for future**			
Communication	**X**	**X**	**X**
Structural changes	**X**	**X**	**X**
Restrictive interventions	**X**	**X**	**X**
Self-management	**X**	**X**	**X**
Assessment	**X**	**X**	
Creative activities		**X**	**X**
Safety measures	**X**		**X**

A tick (**X**) indicates the specific theme is present in the data among a specific group

All groups agreed that it is important to develop communication between patients and nurses. They described how nurses should talk more with patients. Nurses were aware that they should be more cautious when communicating with patients, select their words carefully and consider how to address sensitive topics. Nurses were also aware that they should brief patients on the expectations on the ward (the rules, etc.), respond to patients’ questions, and explain the current situation and coming procedures and treatment processes early enough. The status of newly admitted patients especially worried patients: how could they learn all the regulations and requirements of the ward? In this task, patients proposed more active role for nurses. Informal caregivers simply proposed more communication between patients and nurses regarding the content of the treatment on the ward.

Again, all groups agreed that structural changes on the ward were needed. Nurses proposed that, between themselves, they could have a better team approach. Each ward could offer specialized treatment to different types of patients as they should not be mixed on the same ward. Patients and informal caregivers proposed that aggressive patients should be separated from other patients to calm the ward atmosphere. Each group further proposed that there should be more manpower, fewer patients, and more rooms. Patients felt that ward culture should have fewer routines and more flexibility. Informal caregivers wanted wards to be more attractive and more comfortable, and they thought that everyone should have their own private space.

It was perceived by all groups that restrictive interventions are needed to manage aggressive events. These interventions could be using a table to restrain patients in a sitting position, two staff members standing on both sides of a patient, or patients being restrained in a bed. Patient medication could also be adjusted, or injections used. Appropriate distance should be kept from patients. To avoid aggressive events, the groups valued self-management skills, including nurses calming themselves down, patients learning to ignore noise makers and control themselves, and patients trying not to provoke themselves.

Only nurses and patients described how risk assessment should be developed. Nurses suggested better alarm systems, and that they should be more cautious about possible crises. Nurses felt they should better identify patients’ needs and what makes them angry. Patients proposed that nurses should observe patients, especially recently admitted ones, and be cautious in predicting a patient’s intention. Only patients and informal caregivers described problems on the ward such as a lack of activities, and ‘empty’ days. They suggested outdoor exercises, education, and creative activities. Alternatively, nurses proposed a number of different safety measures to manage patient aggression (e.g., security guards, police), as informal caregivers proposed that it is important to seek dangerous objectives.

## Discussion

This research study is the first to provide a description and comparison of perceptions regarding patient aggression using three stakeholder groups in an Asian country: nurses, patients, and informal caregivers. In the thematic analysis of the focus group interviews with a total of 94 participants, we described aggression events, their possible reasons and outcomes, and ideas for future development of aggression event management. All three groups in our study including patients shared the same understanding of aggression. The description of what happened, what types of aggressive events occurred (physical, verbal, threat) and what was the target of the aggressive events (other people, objects, patients themselves) are all in line with definitions of aggressive events in international literature [5–7]. All stakeholder groups also agreed that any action or intention of aggression aimed to harm something or someone. The finding of mutual understanding between groups gives a good starting point for future development of aggression prevention in psychiatric inpatient hospital care.

Patients and especially nurses described how patient aggression occurred with no clear reason or forewarning. The finding was surprising as to our knowledge, the systematic monitoring of patients’ mental status and risk assessment was already a part of the daily procedures of nurses in all of our study wards. At the same time, all groups agreed that a patient’s poor mental status could be a source of aggression [5]. On the other hand, the mental status of a patient may change rapidly, which may explain why aggressive events were perceived as unpredictable by both nurses and patients. Despite mounting evidence in the international literature, we may only speculate why risk assessment methods have not increased nurses’ confidence to predict patients’ aggressive behavior in psychiatric wards. Just administering an instrument is not a risk assessment [39]. Calculation of a risk estimate is not doing decisions for nurses, so more focus on thought processes and risk management may be needed [39].

All three groups in our study agreed that patients’ unmet needs and a lack of communication with staff members were reasons for patient aggressive behaviour. Better communication and self-management activities were also proposed in all three groups to solve these daily problems on the hospital wards. All participant groups also described how the treatment environment itself may be restrictive and increase aggressive tension, not only in patients [19] but also in nursing staff. Previous studies have shown that working for a long time in a confined environment with a high incidence of aggression may increase nurses’ cynical attitudes towards the treatment system and thereby affect nurses’ behavior [40]. On the other hand, the majority of nurses in our study were junior staff as 53% were between 18 and 35 years old. It has also been shown that less working experience and a lack of confidence in managing challenging situations may increase pressure in nurses [41]. Therefore, training efforts for staff members need to ensure ethically sensitive treatment for patients in hospital wards.

Our study showed that not only nurses but also informal caregivers and patients themselves proposed that restrictive interventions are needed to manage aggressive events. Previous studies have shown that especially nurses’ believe that patients experience therapeutic benefits from the use of coercive measures, which may cause nurses to request more restrictive interventions for agitated patients [42]. Indeed, aggressive vents in our data were typically followed by escalation and the use of physical restrictions, which was seen a normal procedure based on our study results. As an outcome of aggressive events, only nurses and patients expressed experiencing physical burden, while all groups considered psychological burden to be a consequence of aggression. As a contradictory finding, all groups proposed that helpful attitudes among nurses, better communication, structural changes, and better self-management skills would prevent patient aggression.

We also found some sharp contrasts in themes between groups. Patients and informal caregivers described patients on the ward as bored and lacking activity and they proposed more activities on the ward, such as education and outdoor exercises. The nurses did not share this view. On the contrary, nurses emphasized security issues in patient care, such as having more guards and safety measures. Similarly, a Finnish study found that the development of aggression management in nurses’ work focused on better camera surveillance and personal alarm systems [43]. Nurses may currently prioritize the management of aggressive events in their work, whereas patients and families focus on the origins of aggression and how to prevent aggressive events. As nurses are typically a target of patient aggression, balancing nurses’ own safety and patients’ recreational activities makes aggression prevention even more challenging. This apparent dissonance in nurses’ roles may not leave space for the development of creative activities that would improve the ward atmosphere and support positive treatment culture. All groups still mutually described crowded wards as a source of aggressive situations [5], and therefore the development of physical and emotional aspects of treatment environments could be a common area of development in psychiatric hospitals in the future.

### Limitations

Despite of the robust methodology used, our focus group study has limitations that need to be taken into consideration. First, due to safety reasons, some staff members were present in the focus group interviews, which may have influenced the conversations. A second limitation may be that we were working with two languages (Cantonese, English); differences in expressions and meanings between the languages may have affected the data categorization process. The third limitation is related to the study context as people from an Asian culture might have different expectations or acceptance levels of patient aggression and use of coercive methods, thereby leading to some themes being heavily emphasized or overlooked. The results, therefore, may not be generalizable across different cultural contexts. Fourth, we did not include psychiatrists in the focus groups session despite their important role in patient care. The reasons for excluding psychiatrists relate to practical issues and concerns that including psychiatrists with other professional staff in focus groups might stifle discussion due to a strong professional hierarchy in Hong Kong. It was also not feasible to facilitate focus groups specifically for psychiatrists due to their low numbers in the clinical settings. However, in the future it is important that studies engage different stakeholder groups including psychiatrists, social workers, policy makers or any other groups to discuss and share about ideas how to support patient-centred care.

In addition, all participants in the patient groups were invited from closed in-patient wards that support people with severe and enduring mental illnesses and it could be argued that their acute symptoms and potentially impaired cognitive functioning may have influenced their engagement or ability to recall events. We may also speculate whether the results would be different if we would run individual interviews with our vulnerable due to sensitive research topic. For example, a study conducted in Denmark found that out of 47 patients within a heterogeneous diagnostic sample, eight patients lacked any memory of their physical restraint, and the memories of 36 patients could be questioned [44]. We may therefore ask how relevant it is to interview patients in groups who may present cognitive deficits and a lack of ability to share valid perceptions regarding aggression. However, a modern research and treatment approach supports our decision to value patients’ perceptions and decisions in mental health services, despite their mental status or diagnosis [45]. Especially recent literature about shared decision-making supports staff and patient collaboration in practicing and developing aggression risk assessment and management methods [46]. Therefore, this study is a forerunner in Hong Kong and perhaps also in the wider Asian area to listen patient voices and value their perceptions of this complex and sensitive topic.

### Implications of the results in the international context

Our study results offer a novel but complex diversity of perspectives from different stakeholder groups regarding patient aggression. The qualitative approach provided insight into the topics that matter to those persons who are closely involved, either physically or emotionally, to aggressive events in psychiatric hospitals. The findings of the study reflect the results of a document analysis from Hong Kong, which revealed that the local hospital guidelines may not support international standards regarding the reduced use of restrictive interventions [37]. Therefore, understanding how the inpatient culture plays a role in shaping a dynamic between patients and staff and developing communication skills that acknowledge this may help reduce violence and aggression in inpatient settings [10].

Contrary to general assumptions, the participants in our focus groups shared a similar understanding of the phenomenon of aggressive behaviour. This is a good starting point for the future development of positive communication culture between stakeholder groups. Our findings suggest that patient aggressive events may be triggered by ignoring patients’ needs due to a lack of communication skills among nurses [5]. Various methods, such as debriefing after challenging situations, has already been recommended in different treatment guidelines [47, 48]. However, even if the monitoring data of aggressive events might be available, staff may lack the ability to analyse challenging situations more generally, especially from patients’ perspectives. Organising regular meetings with patients could improve staff members’ understanding of patients’ perspectives. This study confirms that nurses, patients, and informal caregivers are able and willing to share their views regarding sensitive issues, and therefore focus groups might be useful method to be used more widely in quality improvement activities in many healthcare settings. This simple, relatively low-cost, and easy-to-use focus-group approach is still uncommon in psychiatric services globally but might be worth testing in different treatment settings, patient groups and countries. The next step could be to form multi-stakeholder groups on psychiatric wards as part of treatment practices to identify common problems and find solutions in treatment practices together.

Our study results indicated that family members were not fully aware of what happens on the ward. This is not surprising since they are only able to visit patients during a short period of time outside of the ward environment. It is therefore important to share general information of patient treatment practices on the wards to improve their awareness of what is happening to their loved-ones. The engagement of informal caregivers in patient care and listening to their opinions are important elements of high quality care, and may help improve patient treatment outcomes [49]. Therefore, we strongly support the argument of Chieze et al. [50] that coercive measures used after a transparent, carefully balanced evaluation process are more likely to be adequate, understood, and accepted by patients and caregivers.

This study primarily offers new insight into patient aggression from perspectives of the various stakeholders. Including participants who have lived experiences of patient aggression, such as nurses and informal caregivers, confirms the value of the approach of engaging all relevant stakeholders to patient care. Based on our experiences of this study, we suggest that this study approach could be feasible for other international settings, as patient aggression is a common problem in any healthcare setting [51]. One strength of the study is the diversity of participants, which resulted in insight from different perspectives and therefore yielded a richer and more complex understanding of the research data.

However, patient safety in inpatient mental health settings is still under-researched in comparison to non-mental health inpatient settings [52]. As the evidence focused on organizational interventions for preventing and minimizing aggression is still of low-quality, more studies in this area are needed [53]. A recent review proposed that to prevent patient aggression in healthcare organizations, the approaches should include multicomponent interventions, ideally involving all stakeholders [54] Our study showed that it is feasible to involve different stakeholders in joint initiatives to reach a shared understanding of the current situation and identify common problems, which might help to make new approaches more acceptable to different parties.

Putting together, our study results offer an additional panorama of insights to the international literature, which can help policy makers (hospital leaders, healthcare practitioners, researchers) to better understand patient aggression in inpatient settings and the main issues stakeholders face. This study also confirms that, regardless of the location, prevalent culture or educational background of respondents, aggression, its consequences, and the related proposed development ideas, seem to generally remain the same.

## Conclusions

Despite of the mounting research, understanding patient aggression in psychiatric hospital settings is still nonconclusive. Real steps are needed to develop appropriate interventions based on the needs identified in nurses’, patients’, and informal caregivers’ perceptions in themes compiled in the study results. Future research should, in the most effective and humane ways, further investigate the impact of the management of patient aggressive behaviour on psychiatric care.

## Supplementary Information

**Additional file 1.**

## Data Availability

The datasets generated and analysed during the current study are not publicly available due confidentiality reasons but are available from the corresponding author on reasonable request.
