# Supplementary material for: Perceptions of patient aggression in psychiatric hospitals: a qualitative study using focus groups with nurses, patients, and informal caregivers
Source: BMC Psychiatry. 2022 May 18;22:344. doi: 10.1186/s12888-022-03974-4 (PMC9118596; doi:10.1186/s12888-022-03974-4)
Supplement: Supplementary file 1 — Additional file 1. [file 12888_2022_3974_MOESM1_ESM.docx]

**PROTOCOL FOR FOCUS GROUP INTERVIEWS**

**BACKGROUND**

Focus groups are designed to obtain the participants’ perceptions in a focused area in a setting that is permissive and nonthreatening. One of the assumptions underlying the use of focus groups is that group dynamics can assist people to express and clarify their views in ways that are less likely to occur in a one-to-one interview. Focus groups should include 8 -10 participants.

**FOCUS GROUP GUIDE**

The participants will be made aware of the time and venue of the focus group. The inclusion criteria for the focus groups are as follows:

- Adults (18 years or above)
- Both gender
- Willingness to participate and share their views in group (or individually if group interview is not possible because of any reason)
- Accept the audio recording

**PREPARATION OF THE FOCUS GROUP INTERVIEWS**

- Organise a comfortable and quiet place for about 1 hour to conduct the interviews (1 extra hour to prepare the interviews and for cleaning the room after the interviews), totally about 2 hours
- Check equipment for audio recording (extra batteries are always useful) and make sure that all participants can be clearly heard on the recording.
- Provide a paper copy of the following to each participant:
  - Background information forms (x 1)
  - Informed consent forms (x2; one copy to be returned to researcher, and another copy to be kept by participant)
  - Written information about the study (x1)
- Make sure that the environment is generally quiet (turn off cell phones etc.)

**FACILITATOR ROLES AND RESPONSIBILITIES**

- One facilitator (lead facilitator) should be identified beforehand. His/her role is to take the lead in asking questions, encouraging participant responses and exploring issues in more depth. This facilitator should practice using the interview schedule prior to the focus groups in order to be familiar and comfortable with the line of questioning.
- A second facilitator (co-facilitator) is responsible for ensuring the equipment is working, keeping the focus groups on time and making observation notes.
- The co-facilitator should also make notes of the participant study identifier number each time they speak.

**BEGINNING OF THE INTERVIEWS**

- Participant will complete the Background question form (anonymous)
- **Participants will be informed about the following:**
  - Background of the study
  - Purpose, aims and objectives of the interview
  - What are the expected results, and how the participants can get information about the results
  - What happens if they participate the interview
  - Information about the benefits and potential risks of the interview
  - Information about the ethical aspects of the interview/study
  - Tell the participants that everything is fully voluntary. They have the right to refuse to participate at any stage of the study and may leave the interview without giving a reason
  - The purpose of tape recording and that confidentiality will be maintained
  - If someone declines to take part, make the refusal situation as easy as possible for the person
  - Information about the roles of participants during the interview
  - Timetable (the interview discussion may take 45 minutes – 2 hours, depending on the participants)
  - Information about the results and reporting of the project
  - Make sure that the participants can also ask questions after the interview, give your contact information for any further questions that may arise
- Written consent form (2 copies) should be signed before the interview by the participants and facilitator
- One signed form will be returned to each participant
- Interview questions should not be given to the participants.

**FOCUS GROUP FORMAT**

All participants, including two focus group interview facilitators, will sit in a circle. Facilitators should sit apart from each other. Participants can freely decide where they want to sit. Focus group interviews should take place in a quiet, comfortable, and private environment.

Interview schedule will be the same for each participant group (patients, nursing staff, and relatives) and following general questions will be asked in each group.

**Introduction**

*“Thank you for coming and joining us today. The reason why we asked you to join this interview today is because we are very interested to hear what your views about violence or aggression in psychiatric hospitals.”.*

*To get started, we are first going to do a little activity to give us a chance to get to know each other. In addition, I would like to ask you to fill in a short background information form – without your full name.”*

Facilitators may ask participants to go around in the circle to briefly introduce themselves. Participants can use nick names.

**INTERVIEW QUESTIONS**

Interview questions will be open-ended, allowing participants to describe their experiences, and ideas on study themes in their own words.

- *Please, identify and describe situations related to patient aggressive behaviour on the ward – what happened?*
- *What might cause the situations?*
- *What are the outcomes of the situation?*
- *How patient aggressive behaviour could be prevented or managed in the future?*

**AT THE END OF THE INTERVIEWS**

- Give chance for extra questions
- Go through participants’ emotions related to interview
- Thank the participants
- Tell once again about the expected results and how the participants are informed about the results

In case of any emotional or negative feelings exist after interviews, the participants are encouraged to share them with the researchers. In addition, contact information with unit personnel or outside counselor will be shared to discuss the emotions further.
